# Early Adverse Effects of Behavioural Preventive Strategies During the COVID-19 Pandemic in Germany: An Online General Population Survey

**DOI:** 10.32872/cpe.7205

**Published:** 2022-09-30

**Authors:** Michael Witthöft, Stefanie M. Jungmann, Sylvan Germer, Anne-Kathrin Bräscher

**Affiliations:** 1 Department of Clinical Psychology, Psychotherapy, and Experimental Psychopathology, Johannes Gutenberg University of Mainz, Mainz, Germany; Philipps-University of Marburg, Marburg, Germany

**Keywords:** quarantine, social, physical distancing, anxiety, depression, somatic symptoms

## Abstract

**Background:**

Quarantine and physical distancing represent the two most important non-pharmaceutical actions to contain the COVID-19 pandemic. Comparatively little is known about possible adverse consequences of these behavioural measures in Germany. This study aimed at investigating potential early adverse effects associated with quarantine and physical distancing at the beginning of the countrywide lockdown in Germany in March 2020.

**Method:**

Using a cross-sectional online survey (N = 4,268), adverse consequences attributed to physical distancing, symptoms of psychopathology, and sociodemographic variables were explored in the total sample as well as in high-risk groups (i.e., people with a physical or mental condition).

**Results:**

The most frequently reported adverse effects were impairment of spare time activities, job-related impairment, and adverse emotional effects (e.g., worries, sadness). Participants with a mental disorder reported the highest levels of adverse consequences (across all domains) compared to participants with a physical disease or participants without any mental or physical condition. No significant association between the duration of the behavioural protective measures and the severity of adverse mental health effects was observed.

**Conclusion:**

Results showed that non-pharmaceutical actions were associated with adverse effects, particularly in people with mental disorders. The findings are of relevance for tailoring support to special at-risk groups in times of behavioural preventive strategies.

## Highlights

- Physical distancing and quarantine were associated with negative psychological effects.
- The most frequently affected areas were spare time activities, job, and emotional condition.
- Participants with a mental disorder reported the highest levels of adverse consequences.
- No significant relation between duration of the protective measures and severity of adverse effects.

## Background

Behavioural non-pharmaceutical interventions and preventive strategies (i.e., isolation, quarantine, and physical distancing) represent the most important first-line interventions to counteract novel pandemics such as COVID-19. Despite its effectiveness, already findings from earlier pandemics suggest that behavioural preventive strategies have psychological costs (e.g., Brooks et al., 2020; Henssler et al., 2021). Similar findings were observed in meta-analyses related to COVID-19 which found small positive associations between the implementation, duration, and stringency of behavioural measures and symptoms of mental disorders (e.g. Jin et al., 2021; O’Hara et al., 2020; Wang et al., 2021). However, another meta-analysis using longitudinal data suggests that the psychological impact of behavioural measures (e.g. lockdown) is weak and heterogenous at best (Prati & Mancini, 2021), and one meta-analysis comparing countrywide point prevalences of depression and stringency levels regarding early interventions (e.g. countrywide lockdowns) found less severe adverse mental health consequences associated with more stringent early interventions (Lee et al., 2021). Due to the heterogeneity of existing findings, this study aimed at investigating possible adverse effects associated with different behavioural preventive strategies (quarantine and physical distancing), particularly during the early stage of the COVID-19 pandemic in Germany in March and April 2020.

Shortly after COVID-19 was declared a pandemic by the WHO on March 11^th^ 2020, preventive actions were taken by the German government and the federal states. Since March 16^th^, federal states decided to close kindergartens and schools and the federal government restricted cross-border traffic from a number of neighboring countries. On 23^rd^ of March, a nationwide assembly ban was established, prohibiting assemblies of more than two persons (except people and families living in the same household). Additionally, restaurants and businesses concerned with body care were immediately closed (Robert Koch Institut [RKI], 2020a), resulting in a partial nationwide lockdown. Despite their effectiveness, comparatively little is known about possible psychological side effects of these preventive actions. Studies from Germany (Benke et al., 2020), Italy (Fornili et al., 2021), the UK (Fancourt et al., 2021), U.S. (Daly & Robinson, 2021), and China (Gan et al., 2022) suggest that government restrictions on daily life (e.g., lockdown and stay-at-home orders) result in significantly elevated levels of psychological distress (mainly increased symptoms of anxiety, depression, and higher levels of loneliness) at the beginning of the pandemic in March 2020. Longitudinal population-based studies in the UK (Fancourt et al., 2021) and U.S. (Daly & Robinson, 2021) suggest that after an initial increase in mental distress during the first wave of the pandemic in March 2020, distress levels significantly declined on the population level, despite continued behavioural restrictions and lockdown measures. It therefore remains unclear, to what extend the observed higher levels of mental distress are directly (i.e., causally) attributable to behavioural preventive strategies. Interestingly and rather unexpectedly, no direct evidence of a dose-response relationship between the intensity (i.e., duration) of the behavioural preventive strategies and levels of psychological distress could be observed, neither in a study from China (Gan et al., 2022) nor an early German study (Benke et al., 2020). Moreover, observed associations between behavioural restrictions and mental distress appear small in terms of effect sizes (Benke et al., 2020; Prati & Mancini, 2021). Gan et al. (2022) interpret this observation as a “psychological typhoon eye effect”, i.e., during an immediate threat, the negative emotional response to a disaster might appear atypically weak at first glance. Alternatively, these findings might suggest that the threat by the disease itself, rather than behavioural precautions might be responsible for the observed adverse mental health effects.

When considering adverse effects of behavioural precautions, three types of strategies have to be conceptually distinguished: (a) isolation (i.e., separation of already infected and thus potentially contagious individuals); (b) quarantine (i.e., separation of individuals with contact to potentially contagious individuals); and (c) social distancing/physical distancing (i.e., restricting social physical contacts as a primary preventive strategy to reduce the number of new infections in the population). Early reviews and meta-analyses suggest adverse mental health effects associated with isolation and quarantine in terms of increased levels of anxiety, depression, and stress (Jin et al., 2021; Wang et al., 2021) and those findings appear similar to results from earlier pandemics as e.g. SARS-CoV or MERS-CoV (e.g., Brooks et al., 2020; Henssler et al., 2021). Still, empirical evidence directly related to different behavioural measures in the COVID-19 pandemic is comparatively sparse. Moreover, earlier reviews and meta-analyses mainly focus on the effects of isolation and quarantine, rather than more general social and physical restrictions that are characteristic of the global response to the COVID-19 pandemic.

The primary aim of this study was to explore the early psychological effects of the most important behavioural non-pharmacological interventions (i.e., physical distancing and quarantine) initiated against the COVID-19 pandemic in Germany in March 2020. Furthermore, this study aimed at examining whether potential high-risk groups within the general population (i.e., people with a current mental disorder or physical disease) were more negatively affected by these actions compared to healthy people without a current mental or physical condition. Finally, it was hypothesized that significant positive dose-response relationships would exist between the duration of the respective behavioural actions (i.e., lockdown, physical distancing, and quarantine) and individual levels of psychological distress or adversities, suggesting first evidence of a causal relationship between the duration of preventive actions and psychological distress levels.

## Method

### Sample and Procedure

The online survey took place between 25^th^ of March and 13^th^ of April 2020, at an early stage of the virus outbreak in Germany, and was presented in German language. The first cases of SARS-CoV-2 infection in Germany became known at the end of January 2020. On March 25^th^, about 31,554 cases of SARS-CoV-2 infection, including 149 deaths (worldwide: 413,467 infections), and on April 13^th^ about 123,016 cases, including 2,799 deaths (worldwide: 1,773,084 infections) were registered (RKI, 2020a, 2020b; WHO, 2020a, 2020b).

Participants were recruited via social media (e.g., Twitter), e-mail distribution lists of student councils at universities, and our department's website. In addition to information on the study (type, content, duration, lottery of gift vouchers as compensation for participation), the study announcements included a link to the online study. Inclusion criteria were a minimum age of 16 and informed consent. The study protocol was approved by the local ethics committee.

Altogether, 4288 persons completed the survey. Twenty persons were excluded due to the following reasons: implausible indication of age (*n* = 2), very fast completion of the questionnaire (*n* = 3), long quarantine (> 33 days) for reasons other than SARS-CoV-2 (*n* = 6), long period (> 50 days) of social distancing (*n* = 9) possibly for reasons other than SARS-CoV-2. The final sample consisted of *N* = 4268 persons (Table 1). Of the participants, 10.5% (*n* = 449) reported to be in quarantine themselves (for *M* = 9.86 days, *SD* = 3.83 [range: 2-30]), 27.1% (*n* = 1156) reported to know someone in their close social environment (family/friends) and 34.0% (*n* = 1451) in their wider social environment (e.g., acquaintances or at the same residence) who had been in quarantine. Concerning physical distancing, participants reported to practice it for an average of 11.85 days (*SD* = 5.18, range [0, 50]). While 0.6% (*n* = 25) reported not reducing their physical contacts at all, 1.7% (*n* = 71) reported to reduce their physical contacts a little, 3.8% (*n* = 161) a medium amount, 23.5% (*n* = 1005) considerably, and 70.4% (*n* = 3,006) very strongly.

**Table 1:** Sample Characteristics (n = 4268)

Variable		
	*M*	*SD*
Age	32.89	12.07
	*N*	%
Sex		
Female	3389	78.9
Male	886	20.8
Diverse	13	0.3
**Born in Germany**	4015	94.1
Professional status		
Employed	1698	39.8
Students	1286	30.1
In school/vocational training	209	4.9
Public servants	209	4.9
Self-employed	204	4.8
Unemployed	145	3.4
Retired	134	3.1
On parental leave	132	3.1
Housewife/househusband	96	2.2
Other	158	3.7
Education		
College/university degree	1866	43.7
General qualification for university entrance	1662	39.0
General Certificate of Secondary Education	529	12.4
Basic school education	117	2.7
Still in school/dropped out of school	74	1.7
Health status		
Healthy	2877	67.4
Physical disease	817	19.1
Psychological disorder	331	7.8
Physical disease and psychological disorder	243	5.7

### Measures

#### Somatic Symptom Reporting

The Patient Health Questionnaire Somatic Symptom Scale (PHQ-15; Kroenke et al., 2002) is a self-administered instrument that assesses the severity of fifteen common somatic symptoms on a scale from 0 (*not bothered at all*) to 2 (*bothered a lot*) covering the preceding four weeks. The PHQ-15 has shown good reliability and validity in previous studies (Gräfe et al., 2004; Kroenke et al., 2002; van Ravesteijn et al., 2009). In the current study, the internal consistency was Cronbach’s α = 0.80.

#### Psychosocial Stress

The Stress Module of the Patient Health Questionnaire (PHQ-Stress; Gräfe et al., 2004) assesses psychosocial stressors (including health, work/financial, social, and traumatic stress) that provide indications of potentially causing or maintaining factors of mental disorders. It is a self-report questionnaire and consists of ten questions referring to the last month, which can be answered on a scale ranging from 0 (*not bothered at all*) to 2 (*bothered a lot*). A limited number of studies suggest adequate reliability and validity of the questionnaire (Beutel et al., 2018; Klapow et al., 2002). Internal consistency in the present study was Cronbach’s α = 0.69.

#### Anxiety and Depression

The Patient Health Questionnaire Depression and Anxiety Screener (PHQ-4; Kroenke et al., 2009) is an ultra-brief screener for anxiety and depression. It is a composite instrument that consists of two items assessing the core criteria for depression and two items assessing core aspects of general anxiety disorder. The scale ranges from 0 (*not at all*) to 3 (*almost every day*) and refers to the last two weeks. Adequate reliability and validity have been demonstrated (Kroenke et al., 2009; Löwe et al., 2010). The internal consistency in this study was Cronbach’s α = 0.84.

#### Loneliness

The three-item loneliness scale (UCLA-LS-3; Hughes et al., 2004) is the short version of the UCLA-loneliness scale (Russell et al., 1980) and assesses subjective isolation. Items can be answered on a scale from 1 (*hardly ever*) to 3 (*often*). Some evidence confirms sufficient reliability and adequate validity of the questionnaire (Hughes et al., 2004). For the current study, the authors translated the three items to German. Internal consistency in the current study was Cronbach’s α = 0.74.

#### Strains/Changes Due to Social/Physical Isolation

In order to assess changes and strains due to the pandemic in more detail, participants were asked whether they experienced the following due to social/physical isolation: more socially isolated/lonely, being separated from important people, lack of leisure activities (e.g., sport), occupational restrictions/job loss, increased computer/internet use, increased TV consumption, more conflicts at home, worsened mood/sadness, worries, anger, boredom, or other. Participants were also asked to indicate how much they felt distressed by the applicable changes/strains on a scale from 1 (*not distressing at all*) to 101 (*extremely distressing*).

### Quantifying the Duration of Quarantine and Physical Distancing

First, the duration of current quarantine and the reduction of social (physical) contacts were assessed via two questions (i.e., with an open-ended response format: For how many days have you been in quarantine? For how many days have you been limiting your social contacts?). As an additional, objective criterion for the duration of physical distancing, we computed the number of days since the official lockdown in Germany (23^rd^ of March 2020).

### Statistical Analyses

Analyses were conducted using SPSS 23 (IBM Corp., 2015) and JASP 0.13 (JASP Team, 2022). For all tests, the alpha level was set to 5%. Eta-squared (η^2^) was calculated as effect size for ANOVAs (η^2^ ≥ 0.01 small effect; η^2^ ≥ 0.06 medium effect; η^2^ ≥ 0.14 large effect) and Cohen’s *d* for (post-hoc) *t*-tests (*d* ≥ 0.30 small, *d* ≥ 0.50 medium, *d* ≥ 0.80 large). For correlation analyses, effect size conventions are *r* ≥ |.10| small; *r* ≥ |.30| medium, *r* ≥ |.50| large (Cohen, 1992). For the corresponding Bayes analyses, Bayes factors (BF) were used to quantify the evidence for H_1_ and H_0_, respectively (e.g. Jarosz & Wiley, 2014; Nuzzo, 2017).

## Results

### Psychological Effects of Behavioural Actions (i.e., Lockdown, Social/Physical Distancing, Quarantine)

#### Strains/Changes Due to Social/Physical Distancing

Of the participants, 1.4% (*n* = 59) did not report any change or distress due to social/physical distancing, 67.4% (*n* = 2875) observed increased computer and/or internet use, 61.7% (*n* = 2632) reported a lack of leisure activities (e.g., sport), 61.5% (*n* = 2624) felt separated from important people, 48.1% (*n* = 2055) reported worries, 44.8% (*n* = 1914) observed increased TV consumption, 42.5% (*n* = 1814) reported occupational restrictions or job loss, 44.2% (*n* = 1886) perceived boredom, 40.7% (*n* = 1735) perceived decreased mood or sadness, 36.9% (*n* = 1574) felt socially isolated or lonely, 17% (*n* = 726) reported to have more conflicts at home, 13.5% (*n* = 578) felt anger, and 12.7% (*n* = 544) noticed other changes or strains. On average, participants experienced 4.91 changes/strains (*SD* = 2.20, range [0, 12]) and they reported an average level of distress of *M* = 54.70 (*SD* = 25.29, range [1, 101]).

### High-Risk Groups With Mental Disorder and/or Physical Disease

#### Perception of Changes/Strains Due to Social Distancing

The four subgroups (i.e., persons with a physical disease, persons with a mental disorder, persons with both a physical disease and a mental disorder, and persons without any reported physical or mental condition) differed significantly in the number of perceived changes/strains and perceived distress due to the changes/strains (Table 2). According to Bonferroni-corrected post-hoc tests, healthy individuals reported as much changes/strains as individuals with a physical disease (*t* = -0.35, *p* > .999, *d* = -0.01) and were similarly distressed (*t* = 1.01, *p* > .999, *d* = 0.04) but reported less changes/strains and were less distressed than individuals with a mental disorder (*t* = -7.31, *p* < .001, *d* = -0.42; *t* = -8.45, *p* < .001, *d* = -0.50) or both a physical disease and a mental disorder (*t* = -5.30, *p* < .001, *d* = -0.35; *t* = -5.34, *p* < .001, *d* = -0.36). Individuals with a physical disorder reported less changes/strains and were less distressed than persons with a mental disorder (*t* = -6.30, *p* < .001, *d* = -0.41; *t* = -8.14, *p* < .001, *d* = -0.53) and persons with both (*t* = -4.66, *p* < .001, *d* = -0.34; *t* = -5.42 *p* < .001, *d* = -0.39). Individuals with a mental disorder did not differ from individuals who had both a physical disease and a mental disorder (*t* = -0.83, *p* > .999, *d* = 0.07; *t* = 1.59, *p* = .675, *d* = -0.14).

**Table 2:** Means Standard Deviations, and ANOVA Results (Frequentist and Bayes) of the Number of Changes/Strains Perceived Due to Social/Physical Distancing and Psychometric Instruments Assessed for the Whole Sample and Different Subgroups

Measures of psychological distress	Total sample *M* (*SD*)	Subgroups				*F* test (*p); BF_inclusion_* post-hoc tests
		1	2	3	4	
		No mental or physical disease *M* (*SD*)	Physical disease *M* (*SD*)	Mental disorder *M* (*SD*)	Physical disease and mental disorder *M* (*SD*)	
Number of changes/strains	4.91 (2.20)	4.79 (2.18)	4.92 (2.13)	5.71 (2.25)	5.56 (2.27)	25.64, (< .001); 1.41*10^13^ 1, 2 < 3, 4
Distress due to changes/strains	54.70 (25.29)	53.43 (24.98)	52.43 (26.02)	65.70 (22.92)	62.34 (24.49)	37.66, (< .001); ∞ 1, 2 < 3, 4
PHQ-15	6.97 (4.71)	5.97 (4.19)	8.05 (4.78)	9.95 (5.16)	11.16 (4.86)	161.49, (< .001); ∞ 1 < 2 < 3 < 4
PHQ-stress	5.75 (3.60)	5.17 (3.37)	6.18 (3.54)	7.93 (3.92)	8.20 (3.70)	100.73, (< .001); ∞ 1 < 2 < 3, 4
PHQ-4	3.66 (2.88)	3.21 (2.64)	3.59 (2.62)	6.12 (3.28)	5.84 (3.19)	125.15, (< .001); ∞ 1 < 2 < 3, 4
UCLA-LS-3	6.08 (1.75)	5.93 (1.71)	5.98 (1.74)	7.09 (1.66)	6.81 (1.82)	60.61, (< .001); ∞ 1, 2 < 3, 4

*Note.* PHQ-15, Patient Health Questionnaire Somatic Symptom Scale; PHQ-stress, Patient Health Questionnaire Stress Module; PHQ-4, Patient Health Questionnaire Depression and Anxiety Screener; UCLA-LS-3, 3-item short version of the UCLA loneliness scale.

#### PHQ-15

The subgroups differed concerning their reporting of somatic symptoms, *F*(3, 680.65) = 161.49, *p* < .001, η^2^ = 0.12. Post-hoc tests indicated that all subgroups differed from each other; healthy individuals had lower scores than persons with a physical disease (*t* = -11.83, *p* < .001, *d* = -0.48), individuals with a mental disorder (*t* = -15.44, *p* < .001, *d* = -0.92), and individuals with both (*t* = -17.50, *p* < .001, *d* = -1.22). Further, individuals with a physical disease showed lower scores than individuals with a mental disorder (*t* = -6.55, *p* < .001, *d* = -0.39) and individuals with both (*t* = -9.58, *p* > .001, *d* = -0.65). Individuals with a mental disorder reported less somatic symptoms than individuals with both a physical disease and a mental disorder (*t* = -3.23, *p* = .007, *d* = -0.24).

#### PHQ-Stress

The subgroups differed with regards to their level of psychosocial stress, *F*(3, 688.77) = 100.73, *p* < .001, η^2^ = 0.08. According to post-hoc tests, healthy individuals had lower stress levels compared to individuals with a physical disease (*t* = -7.34, *p* < .001, *d* = -0.30), a mental disorder (*t* = -13.69, *p* < .001, *d* = -0.80), and both (*t* = -13.06, *p* < .001, *d* = -0.89). Individuals with a physical disease were less stressed than individuals with a mental disorder (*t* = -7.73, *p* < .001, *d* = -0.48) and both (*t* = -7.96, *p* < .001, *d* = -0.56). There was no difference between individuals with a mental disorder and both a physical disease and a mental disorder regarding psychosocial stress level (*t* = -0.92, *p* = .793, *d* = -0.07).

#### PHQ-4

The subgroups significantly differed in the screening for depression and anxiety, *F*(3, 681.77) = 125.15, *p* < .001, η^2^ = 0.11. Healthy individuals had lower scores compared to individuals with a physical disease (*t* = -3.52, *p* = .003, *d* = -0.14), a mental disorder (*t* = -18.40, *p* < .001, *d* = -1.07), and both (*t* = -14.46, *p* < .001, *d* = -0.98). Individuals with a physical disorder scored lower than individuals with a mental disorder (*t* = -14.25, *p* < .001, *d* = -0.90) and both (*t* = -11.31, *p* < .001, *d* = -0.82). Individuals with a mental disorder did not differ significantly from individuals with both a physical disease and a mental disorder (*t* = 1.21, *p* = .621, *d* = 0.09).

#### UCLA-LS-3

The subgroups significantly differed in their perception of loneliness, *F*(3, 4264) = 60.61, *p* < .001, η^2^ = 0.04. Post-hoc tests indicated that healthy individuals did not differ significantly from individuals with a physical disease (*t* = -0.71, *p* = .895, *d* = -0.03), but had lower scores compared to individuals with a mental disorder (*t* = -11.59, *p* < .001, *d* = -0.68) and both a physical disease and a mental disorder (*t* = -7.65, *p* < .001, *d* = -0.51). Individuals with a physical disease had lower scores than persons with a mental disorder (*t* = -9.89, *p* < .001, *d* = -0.64) and individuals with both (*t* = -6.61, *p* < .001, *d* = -0.47). No significant difference occurred between individuals with a mental disorder and both a physical disease and a mental disorder (*t* = 1.91, *p* = .224, *d* = 0.16).

### Associations Between Sociodemographic Factors and Perceived Changes/Strains (Number of Strains and Perceived Distress) Due to Physical Distancing

The results of (frequentist and Bayesian) multiple regression analyses (Table 3) suggest that the number of strains attributed to physical distancing was significantly (and independently) associated with lower age, being female, lower educated, living alone, having a current mental disorder, and having a current physical disease. Similarly, perceived distress of physical distancing was significantly (and independently) associated with the same factors, except for the presence of a current physical disease (Table 3).

**Table 3:** Associations (Multiple Regression) Between Sociodemographic Factors and Perceived Changes/Strains (Number of Strains and Perceived Distress) Due to Physical Distancing (N = 4171)

Predictor variables	Dependent variables							
	Number of physical distancing strains (0 – 12)				Perceived distress of physical distancing (0 – 100)			
	*B*	*SE(B)*	β	*p*	*B*	*SE(B)*	β	*p*
Age	< -0.04	< 0.01	-0.21^b^	< .01	-0.24	0.04	-0.11^b^	< .01
Sex (1 = female; 2 = male)	- 0.37	0.08	-0.07^b^	< .01	-4.41	0.94	-0.07^b^	< .01
Education (1 = low; 2 = medium; 3 = high)	-0.24	0.06	-0.06^b^	< .01	-4.61	0.68	-0.10^b^	< .01
Currently unemployed (1 = yes; 0 = no)	0.12	0.19	0.01^e^	.53	0.56	2.17	< 0.01^e^	.80
Living alone (1 = yes; 2 = no)	-0.21	0.09	-0.04^e^	.02	-3.57	1.01	-0.06^b^	< .01
Children (1 = yes; 0 = no)	0.09	0.08	0.02^e^	.27	4.22	0.91	0.08^b^	< .01
Current mental disorder (1 = yes; 0 = no)	0.72	0.10	0.11^b^	< .01	9.74	1.15	0.13^b^	< .01
Current physical disease (1 = yes; 0 = no)	0.22	0.08	0.04^a^	.01	-0.26	0.92	< 0.01^e^	.78
*R^2^*	.07 (*p* < .01)^b^				.05 (*p* < .01)^b^			

*Note.* Results of independent Bayesian regression analyses: ^a^BF_inclusion_ / BF_10_ = 3 - 10 (moderate evidence for H_1_), ^b^BF_inclusion_ / BF_10_ > 10 (strong evidence for H_1_), ^c^BF_inclusion_ / BF_10_ = 1/10 - 1/3 (moderate evidence for H_0_); ^d^BF_inclusion_ 7 BF_10_ = 1/30 – 1/10 (strong evidence for H_0_); ^e^weak/inconclusive evidence.

### Associations Between Behavioural Actions (Quarantine and Physical Distancing) and Levels of Psychological Distress

Correlation analyses (Table 4) suggest that current behavioural actions (quarantine and physical distancing) are weakly positively associated with symptoms of stress, anxiety, and depression (PHQ) as well as somatic symptoms (PHQ-15) and loneliness (UCLA-LS-3). The corresponding Bayes factors (BF) suggest moderate to strong evidence for a positive relationship in all but one of the associations (in case of stress and physical distancing; BF_10_ = 0.55 indicating inconclusive evidence for a relationship).

**Table 4:** Associations Between Behavioral Actions and Measures of Psychological Distress

Measures of psychological distress	Quarantine, currently at the day of assessment (1 = no; 2 = yes)	Strength of physical distancing, currently (1 = no to 5 = extremely)	Days since official lockdown in Germany (23.03.2020)	Self-reported duration physical distancing (days)	Self-reported duration quarantine (days)^‡^
Stress (PHQ)	.06*^b^ (.06*)	.04^e^ (.03)	-.04*^c^ (-.04*)	.01^d^ (.02)	-.09^e^ (-.09)
Anxiety/ Depression (PHQ-4)	.06*^b^ (.04*)	.06*^b^ (.06*)	<.01^d^ (.02)	.04^c^ (.06*)	-.04^d^ (-.02)
Somatic symptoms (PHQ-15)	.09*^b^ (.08*)	.05*^a^ (.04*)	-.03^c^ (-.02)	.04^c^ (.05*)	-.08^c^ (-.05)
Loneliness (UCLA-LS3)	.07*^b^ (.05*)	.09*^b^ (.10*)	<-.01^d^ (.01)	-.03^c^ (-.02)	-.02^d^ (-.01)

*Note.* Coefficients represent Pearson’s Rho; corresponding partial correlation coefficients conditioned on age, sex and education in parentheses (npartial corr = 4171); results of independent Bayesian regression analyses: ^a^BF_10_ = 3 - 10 (moderate evidence for H_1_). ^b^BF_10_ > 10 (strong evidence for H_1_). ^c^BF_10_ = 1/10 - 1/3 (moderate evidence for H_0_). ^d^BF_10_ = 1/30 – 1/10 (strong evidence for H_0_). ^e^inconclusive evidence. PHQ = Patient Health Questionnaire; PHQ-4 = Patient Health Questionnaire-4 (brief screening for anxiety and depression). PHQ-15 = 15-item somatic symptom subscale of the Patient Health Questionnaire; UCLA-LS3 = 3-item short version of the UCLA loneliness scale; **^‡^**subsample of participants reporting at least 1 day of quarantine (*n* = 449; *n_partial corr_* = 431).

*p < .01.

Further correlational analyses focusing on possible associations between the duration of behavioural actions and levels of psychological distress (Table 4) suggest that the duration (in days) since the start of the lockdown is largely unrelated to symptoms of stress, anxiety and depression, somatic symptoms, and loneliness (with correlation coefficients ranging from -.04 to .001). The evidence in favour of H_0_ (i.e., no association between the respective variables) is thereby moderate (somatic symptoms) to strong (anxiety and depression, loneliness), and inconclusive regarding symptoms of stress (PHQ). Using the self-reported number of days regarding physical distancing resulted in almost equivalent findings: correlation coefficients were very small in size (range: -.03 - .04) with moderate (anxiety and depression, somatic symptoms, loneliness) to strong (stress symptoms) evidence in favour of H_0_ (i.e., no association between the respective variables).

Similarly, the self-reported number of days in quarantine (for the subsample of participants *n* = 449 reporting at least 1 day of quarantine) showed consistently small negative associations (range: -.09 - .02) with symptoms of stress, loneliness, and psychopathology. Bayes factors were indicative of mostly moderate to strong support for H_0_ (i.e., no association exists between the respective variables). In sum, support for a dose-response relationship as evidence of causality between symptom severity and behavioural measures was observed neither for the duration of physical distancing nor the duration of quarantine.

Since the day-wise subsamples differ in terms of sociodemographic variables, we additionally computed partial correlations (with statistically controlling for age, sex, and education) as a robustness check (Table 4). The pattern of correlations remained largely unchanged. Only two of the reported associations reached statistical significance (the association between self-reported days of physical distancing and symptoms of anxiety and depression in the PHQ-4: *r_partial_* = .06, *p* < .01; the association between self-reported days of physical distancing and somatic symptoms in the PHQ-15: *r_partial_* = .05, *p* < .01). The changes in the strength of associations are generally small and not indicative of qualitatively meaningful differences, though.

### Associations Between COVID-19 Anxiety, Strength of Physical Distancing, Symptoms of Stress and Psychopathology, and Perceived Changes/Strains of Physical Distancing

Associations between COVID-19 anxiety, strength of physical distancing, number of COVID-19 cases and subjective measures of distress and psychopathology are detailed in Table 5. COVID-19 anxiety shows significant medium sized associations with symptoms of stress, anxiety, depression, and somatic symptom distress in the PHQ. Self-reported strength of physical distancing showed only small associations with loneliness, the number of strains of physical distancing and associated distress but not with any of the PHQ measures. Neither the number of days since lockdown nor the daily number of COVID-19 cases were significantly associated with symptoms of stress, psychopathology, or loneliness.

**Table 5:** Associations Between COVID-19 Anxiety, Strength of Physical Distancing, Symptoms of Stress and Psychopathology, and Perceived Changes/Strains Due to Physical Distancing

Predictor variables	Dependent variables					
	Stress (PHQ)	Anxiety/ Depression (PHQ-4)	Somatic symptoms (PHQ-15)	Loneliness (UCLA-LS3)	Physical distancing strains (0 - 12)	Distress physical distancing (0 – 100)
COVID-19 anxiety	.34*^b^ (.31*^b^)	.30*^b^ (.28*^b^)	.33*^b^ (.30*^b^)	.19*^b^ (.17*^b^)	.14*^b^ (.14*^b^)	.23*^b^ (.22*^b^)
Strength of physical distancing (1 = no to 5 = extremely)	-.01^d^ (-.02^c^)	.02^d^ (.02^e^)	< -.01^d^ (<-.01^c^)	.07*^b^ (.07*^b^)	.09*^b^ (.09*^b^)	.09*^b^ (.08*^b^)
Days since official lockdown in Germany (23.03.2020)	.15^c^ (.19^c^)	.24^d^ (.19^e^)	.13^d^ (.13^c^)	.24^c^ (.22^e^)	.04^e^ (< -.01^e^)	.19^e^ (.18^e^)
Daily COVID-19 cases (per million)	-.18^c^ (-.21^e^)	-.22^d^ (-.16^e^)	-.15^d^ (.-.14^c^)	-.23^c^ (-.19^e^)	-.07^e^ (.01^e^)	-.23^e^ (-.19^e^)

*Note.* Table contains beta coefficients of multiple regression analyses; corresponding values after adjusting for sex, age, and education in parentheses (*n* = 4171); results of independent Bayesian regression analyses: ^a^BF_inclusion_ / BF_10_ = 3 - 10 (moderate evidence for H_1_). ^b^BF _inclusion_ / BF_10_ > 10 (strong evidence for H_1_). ^c^BF _inclusion_ / BF_10_ = 1/10 - 1/3 (moderate evidence for H_0_). ^d^BF _inclusion_ / BF_10_ = 1/30 – 1/10 (strong evidence for H_0_). ^e^weak/inconclusive evidence.

*p < .01.

## Discussion

The primary aim of this study was to investigate potential early adverse effects associated with behavioural non-pharmacological preventive strategies (i.e., quarantine and social/physical distancing) initiated at the onset of the COVID-19 pandemic in Germany in March 2020. The majority of the studied sample (98.6%) reported significant changes and adverse effects of physical distancing, with restricted spare time activities, job-related difficulties, and negative emotional consequences as the most frequent topics. Regarding potential high-risk groups, people with a mental disorder (regardless of an additional physical health condition) reported significantly higher levels of adverse effects associated with the social restrictions resulting from physical distancing.

Early reviews on potential adverse effects of quarantine and social distancing (e.g., Brooks et al., 2020) suggest a dose-response relationship between the duration of quarantine and social distancing and the burden of adverse psychological effects. In our study, no such evidence for a dose-response relationship emerged, i.e., no meaningful association was observed between the duration of physical distancing (both at the level of self-report and objective assessment) or duration of quarantine and symptoms of psychopathology. The findings suggest that the (causal) association between the duration of behavioural preventive strategies (i.e., quarantine and social/physical distancing) and symptoms of psychopathology might be smaller than expected, although caution must be taken that these observations might be specific to the situation (and particularly the restrictiveness of the measures) in Germany between March, 25^th^ and April, 14^th^. Consequently, increased levels of psychopathology observed in early stages of the pandemic (e.g., Benke et al., 2020) might be stronger related and attributable to the perceived threat by COVID-19, rather than to the behavioural measures imposed to contain the pandemic. In line with this hypothesis, COVID-19 anxiety appears to be stronger related to measures of negative affect and psychopathology compared to the strength of behavioral measures (Table 5).

Overall, our results are in line with a recent meta-analysis focusing on longitudinal and natural-experimental data across Europe, North America, and Asia suggesting that “the psychological impact of COVID-19 lockdowns is small in magnitude and highly heterogeneous, suggesting that lockdowns do not have uniformly detrimental effects on mental health and that most people are psychologically resilient to their effects” (Prati & Mancini, 2021, p. 201). Additionally, the implementation of stringent behavioral measures might not exclusively be associated with more adverse negative mental health consequences but might also serve as a protective factor, not only in terms of physical but also for mental health (Lee et al., 2021). It appears noteworthy that our study focused primarily on physical distancing compared to quarantine. Since the restrictions associated with quarantine appear more stringent and severe, it might be possible that quarantine could have more stable adverse mental health effects compared to physical distancing (e.g. Jin et al., 2021; Wang et al., 2021).

### Strengths and Limitations

The generalization of findings is restricted by the nature of the sample: The current sample represents an online convenience sample and therefore consists of a higher percentage of women, younger people, and people with higher education and socio-economic status compared to strictly population-representative samples. Therefore, two opposing biases might be existent in the data: Women and younger people have been found to report higher levels of mental distress (Bräscher et al., 2021), i.e., these groups might increase the distress levels observed in our study. On the other hand, the underrepresentation of people with lower education and socio-economic status might lower the observed distress levels in our study. It is difficult to determine, which of the two trends is stronger in size, but representative samples are needed to confirm the current results.

Because this study relied on self-reported questionnaire data only, the formation of subgroups regarding the presence of a mental disorders or a physical disorder should be interpreted cautiously, and further studies using clinical interviews are necessary to confirm our findings and to quantify the amount of additional distress associated with different kinds of mental and physical disorders.

Finally, the examination of possible dose-response associations between distress levels and the duration of the respective behavioural intervention is limited by the cross-sectional nature of our study, the comparatively short period of data assessment (over the period of 20 days), and early point in time in the pandemic situation. More extended, longitudinal studies are needed to rigorously test the question of possible dose-response relationships that would be indicative of a causal relation between duration of non-pharmacological interventions and adverse mental health effects.

### Conclusion

This study aimed at evaluating possible adverse effects associated with non-pharmacological preventive measures imposed to contain the COVID-19 pandemic in Germany. The findings suggest that most of the participants were negatively affected by the behavioural interventions with restrictions in spare time activities, occupational problems, and negative emotional reactions (e.g., worries, sadness, and loneliness). The adverse effects were highest in people with a mental disorder, suggesting that this group should receive particular attention and support in order to prevent exacerbations of mental distress levels. Significant positive association (as possible evidence of a dose-response relationship) with mental distress could neither be observed for the duration of physical distancing nor for the duration of quarantine, leaving open the question whether higher levels of mental distress observed during early stages of the first wave of COVID-19 are *causally* related to the behavioural interventions.
